# Policy Series: GSA Congressional Update

**DOI:** 10.1093/geroni/igaf122.1327

**Published:** 2025-12-31

**Authors:** Brian Lindberg, Patricia D’Antonio

## Abstract

This session provides Hill staff perspectives on what has happened in the aging and health care space during the first ten months of the 119th Congress. Of particular importance will be the Older Americans Act Reauthorization, including funding for the Center for Research and Demonstration in the Aging Network, as well as an update on Medicare Advantage, nursing home regulations, and hospice and palliative care legislation.

